# Florfenicol As a Modulator Enhancing Antimicrobial Activity: Example Using Combination with Thiamphenicol against *Pasteurella multocida*

**DOI:** 10.3389/fmicb.2016.00389

**Published:** 2016-03-30

**Authors:** Chia-Fong Wei, Jui-Hung Shien, Shao-Kuang Chang, Chi-Chung Chou

**Affiliations:** ^1^Department of Veterinary Medicine, College of Veterinary Medicine, National Chung Hsing UniversityTaichung, Taiwan; ^2^Graduate Institute of Veterinary Medicine, National Taiwan UniversityTaipei, Taiwan

**Keywords:** antibiotic combination, synergy, florfenicol, thiamphenicol, oxytetracycline

## Abstract

Synergistic effects between the same class of antibiotics are rarely reported. Our previous study found synergistic-like interaction between florfenicol (FFC) and thiamphenicol (TAP) against *Staphylococcus aureus*. Here, the enhanced antimicrobial activity was evaluated in 97 clinical isolates of both Gram-negative and Gram-positive bacteria. Susceptible strains were initially identified by checkerboard microdilution assay (fractional inhibitory concentration index [FICI] ≤ 0.625), followed by confirmation of synergism using the time-kill methodology (≥2 log_10_ CFU/ml reduction). In all, 43% of *Pasteurella multocida* tested were susceptible to the enhanced bactericidal effect. In chicken fowl cholera models, FFC and TAP combination at much lower dosage that is correspondent to their MIC deduction provided maximum protection *in vivo*. Furthermore, synergistic combination of FFC with oxytetracycline (OTC) against *Pseudomonas aeruginosa in vitro* was also demonstrated. Based on the enhanced uptake of TAP and OTC, FFC presumably elicits enhanced antimicrobial activity in an orderly manner through alteration of bacterial membrane permeability or efflux systems and subsequent increase of intracellular concentration of the antibiotics used in combination. Results of ethidium bromide accumulation assay and RNA-seq showed little evidence for the involvement of efflux pumps in the synergy but further investigation is required. This study suggests the potentiality of a novel combination regimen involving FFC as an initiating modulator effective against both Gram-positive and Gram-negative bacteria depending on the antibiotics that are combined. The observed improvement of bacteriostatic effect to bactericidal, and the extended effectiveness against FFC-resistant bacterial strains warrant further studies.

## Introduction

Florfenicol (FFC) and thiamphenicol (TAP), belonging to amphenicols are broad-spectrum antibiotics commonly used in veterinary or aquacultural practice (Campa-Córdova et al., 2005; Dowling, 2013). This kind of antibiotics binds irreversibly to the 50S ribosomal subunit preventing protein synthesis (Dowling, 2013). TAP is a derivative of chloramphenicol (CAP) in which the *p*-nitro group is replaced by a sulfomethyl group whereas FFC substitutes the hydroxyl group at C-3 site with a fluorine atom (Sams, 1995). Unlike CAP, FFC, and TAP were not reported to cause aplastic anemia (Yunis and Gross, 1975). Moreover, FFC is active at lower concentrations than TAP and CAP against a number of bacterial pathogens *in vitro* and against many CAP-resistant or TAP-resistant strains of common infections in domestic animals (Neu and Fu, 1980; Syriopoulou et al., 1981; Graham et al., 1988; Ueda and Suenaga, 1995). In fact, owing to the broader spectrum of activity and lower toxicity, FFC has been a better antimicrobial agent replacing CAP. FFC is increasingly employed in livestock and aquaculture for treating bacterial diseases associated with respiratory pathogens such as *Actinobacillus pleuropneumoniae, Bordetella bronchiseptica, Mannheimia haemolytica, Pasteurella multocida*, and *Streptococcus suis* (Ferreira et al., 2007). In our previous study, more than 90% of FFC was found to be structurally degraded after 2 h of heating in boiling water. However, the reduced FFC concentration maintained the same antimicrobial activity against both *Escherichia coli* and *S. aureus* due to its conversion to TAP (Franje et al., 2010). Therefore, possible synergistic interactions between these two compounds are hypothesized for further investigation in the present study.

Combination therapy is frequently used to provide a broader antibacterial spectrum and to minimize toxicity as well as the emergence of resistant bacteria (Eliopoulos and Eliopoulos, 1988). The synergism between the same class of antibiotics was rare but not unforeseen. The use of dual β-lactam antibiotics carbapenem (Ertapenem-Doripenem) was recently demonstrated to be effective against KPC-producing *Klebsiella pneumoniae* in both *in vitro* and *in vivo* models (Bulik and Nicolau, 2011; Wiskirchen et al., 2013) and in clinical assay (Ceccarelli et al., 2013). Thus, following on our initial lead reported in 2010 (Franje et al., 2010) possible synergistic antimicrobial activity between FFC and TAP is worthy of further investigation. Here, the antimicrobial activity of FFC/TAP combination in 97 clinically isolated pathogens including Gram-negative (*E. coli, Riemerella anatipestifer, Salmonella entrica*, and *P. multocida, Pseudomonas aeruginosa*) and Gram-positive (*S. hyicus, and S. suis*) bacteria and three ATCC standard strains were investigated. *In vivo* synergism against *P. multocida* in chicken were evaluated. Possible mechanisms contributing to the synergistic activity were also explored.

## Materials and methods

### Bacterial strains and culture conditions

A total of 97 clinically isolated strains were obtained from the National Taiwan University Veterinary Hospital and the Veterinary Medical Teaching Hospital of National Chung Hsing University, Taiwan mainly within the past 5 years (Table 1, Table S1). Three standard strains including *E. coli* (ATCC25922), *P. aeruginosa* (ATCC27853), and *S*. Typhimurium (ATCC19585) were purchased from American Type Culture Collection (Manassas). All isolates were identified to the species level using biochemical methods and PCR analysis. For routine culture, *E. coli, P. aeruginosa, S. hyicus*, and *S. entrica* were grown on trypticase soy broth (TSB). *P. multocida, R. anatipestifer*, and *S. suis* were grown on brain heart infusion (BHI). All bacteria were incubated at 37°C. FFC (molecular weight: 358.21) and TAP (molecular weight: 356.22) were purchased from Sigma-Aldrich. Stock of TAP and FFC were dissolved in dimethylformamide (DMF) such that the final solvent concentration in all wells was less than 5% and showed minimal effect on bacterial growth.

**Table 1:** ***In vitro* inhibitory activity of florfenicol (FFC) and thiamphenicol (TAP) alone and in combination against *P. multocida, S. suis*, and *S. hyicus***.

**Strains**	**host**	**MIC (mg/L) of antibiotics alone**		**MIC (mg/L) in combination**	**Fold MIC in combination**		**FICI**
		**FFC**	**TAP**	**FFC + TAP**	**FFC**	**OTC**	
***Pasteurella multocida***							
101-035	Goose	16	512	0.5 + 256	1/32	1/2	0.531
D7	Pig	0.5	1	0.0156 + 0.5	1/32	1/2	0.531
D13	Pig	0.5	1	0.0313 + 0.5	1/16	1/2	0.563
101-086	Goose	16	1024	1 + 512	1/16	1/2	0.563
102-033	Duck	16	1024	1 + 512	1/16	1/2	0.563
A8	Pig	0.5	1	0.0625 + 0.5	1/8	1/2	0.625
D3	Pig	0.5	1	0.0625 + 0.5	1/8	1/2	0.625
D4	Pig	0.5	1	0.0625 + 0.5	1/8	1/2	0.625
0425	Pig	1	1	0.125 + 0.5	1/8	1/2	0.625
0965	Pig	32	512	4 + 256	1/8	1/2	0.625
D14	Pig	0.5	1	0.125 + 0.5	1/4	1/2	0.75
D48	Pig	0.5	1	0.125 + 0.5	1/4	1/2	0.75
101-001	Duck	0.25	512	0.0625 + 256	1/4	1/2	0.75
0037	Pig	1	2	0.25 + 1	1/4	1/2	0.75
0890	Pig	1	2	0.25 + 1	1/4	1/2	0.75
2223	Pig	64	512	16 + 256	1/4	1/2	0.75
A52	Pig	0.5	1	0.25 + 0.5	1/2	1/2	1
D1	Pig	0.5	1	0.25 + 0.5	1/2	1/2	1
D2	Pig	0.5	1	0.25 + 0.5	1/2	1/2	1
D55	Pig	0.5	0.5	0.25 + 0.25	1/2	1/2	1
1580	Pig	64	512	32 + 256	1/2	1/2	1
0104	Pig	0.5	1	0.25 + 0.5	1/2	1/2	1
0025	Pig	32	512	16 + 256	1/2	1/2	1
***Streptococcus suis***							
CY13005-1L	Pig	2	8	0.25 + 4	1/8	1/2	0.625
BL25-2	Pig	2	4	0.25 + 2	1/8	1/2	0.625
CC13002-2	Pig	0.5	2	0.125 + 1	1/4	1/2	0.75
CC13002-3	Pig	2	4	0.5 + 2	1/4	1/2	0.75
CP13021-1	Pig	2	4	0.5 + 2	1/4	1/2	0.75
CY13005-1B	Pig	1	2	0.25 + 1	1/4	1/2	0.75
CY13005-3	Pig	1	2	0.25 + 1	1/4	1/2	0.75
CS13013-1	Pig	2	4	0.5 + 2	1/4	1/2	0.75
CM166-1	Pig	1	2	0.5 + 1	1/2	1/2	1
BL25-1	Pig	2	8	1 + 4	1/2	1/2	1
CY13005-2	Pig	1	2	0.5 + 1	1/2	1/2	1
CH13011	Pig	1	2	0.5 + 1	1/2	1/2	1
BL13014-3	Pig	1	2	0.5 + 1	1/2	1/2	1
***Staphylococcus hyicus***							
CM134-2	Pig	64	1024	4 + 512	1/16	1/2	0.563
CM144-2C	Pig	128	1024	32 + 512	1/4	1/2	0.75
BL11-3	Pig	4	16	1 + 8	1/4	1/2	0.75
BL11-1	Pig	4	16	2 + 8	1/2	1/2	1
BL11-2	Pig	4	16	2 + 8	1/2	1/2	1
CM144-1C	Pig	8	32	8 + 32	1	1	2

### Susceptibility testing

The minimum inhibitory concentration (MIC) determinations were performed according to the broth microdilution method described in the Clinical and Laboratory Standard Institute guidelines (CLSI, 2013). Briefly, serial 2-fold dilutions of FFC or TAP in cation-adjusted Mueller-Hinton II broth (MHIIB; Difco) or MHIIB supplemented with 5% sheep blood for *S. suis* were prepared in a 96-well U bottom microtiter plate (Nunc, ThermoScientific). Bacteria at final concentration of 5 × 10^5^ colony-forming unit (CFU)/mL were inoculated into each well and grown at 37°C for 18 h. The plates were assessed by eye. The MIC was determined to be the lowest concentration with no visible clumps. *E. coli* (ATCC 25922) was used as a reference strain for the standardization of antibiotic susceptibility. All MIC assays in this study were done at least in triplicate.

### Studies of synergistic effects

The synergistic interaction between FFC and TAP was assessed by the checkerboard microdilution method using MHIIB in triplicates in 96-well plates. Prior to the addition of bacteria, serial dilutions of FFC and TAP were made to create different concentration combinations in each well. In cases that the serial dilutions go beyond the capacity of the 96-well plate, a second plate will be used to complete the checkerboard analyses. The bacteria at a final concentration of 5 × 10^5^ CFU/mL was added to each well. The fractional inhibitory concentration index (FICI) was calculated as the sum of the MIC of each compound when used in combination divided by the MIC of each compound used alone (EUCAST, 2000). The obtained results were interpreted in accordance with the BSAC recommendation as follows: synergy (FICI ≤ 0.5); no interaction (FICI > 0.5–4); antagonism (FICI > 4) (Odds, 2003).

### Time-kill study

Time-kill studies to evaluate the killing dynamics of antibiotics and to determine the synergistic effect of drug combination were performed according to the guidelines by the CLSI (NCCLS, 1999). Exponentially growing cultures of tested strains were diluted to ~5 × 10^6^ CFU/mL in MHIIB. Tubes containing 10 mL cultures were exposed to FFC alone at 1 × MIC (see Table 1 for respective concentrations), or TAP alone at 1 × MIC, or FFC plus TAP at 1 × MIC and incubated at 37°C. One tube containing 10 mL culture without antibiotic was used as a growth control. At 0, 2, 4, 8, 12, and 24 h, 20 μL aliquots obtained from tubes were inoculated on TSA, BHI, or blood agar for colony counts after 10-fold serial dilutions. CFU were counted after incubation at 37°C for 24 h. Synergy was defined as an decrease of ≥2 log_10_ CFU/mL resulted by the antibiotic combination compared to that by the more active antibiotic alone at 24 h, while a change of < 2 log_10_ CFU/mL was considered indifferent (Pillai et al., 2005). Bactericidal activity was defined as ≥3 log_10_ CFU/mL reduction (99.9% kill) in colony count from the starting inoculum. The detection limit was 2.7 log_10_ CFU/mL. All CFU counting was done in duplicate.

### Ethics statement

The animal study was approved by the Institutional Animal Care and Use Committee at National Chung Hsing University (IACUC approval No: 101-104^R2^). The animals were handled in strict accordance with the Guide for the Care and Use of Laboratory Animals of the National Institutes of Health. Experimental animals were euthanased using 100% CO_2_ if they met any early removal criteria (Humane endpoints: neural signs or cyanosis) to minimize suffering. In addition, the possibility of animal death without euthanasia due to acute onset of disease (septicemia) was stated for approval by the IACUC.

### Animal study

In order to evaluate *in vivo* synergistic effect of antibiotic combination, a fowl cholera model (Sarközy et al., 2002) was performed in broiler chickens by intramuscular injection of *P. multocida*. Thirty-five 3-week-old broilers (~1 kg in weight) are divided into seven groups with five replicates each. Chickens had *ad libitum* access to feed and water throughout the experiment. The bacterial inoculum was prepared from an overnight culture of *P. multocida* (101-035) and reconstituted in phosphate-buffered saline (PBS). A dilution solution (25% 2-pyrrolidone) was used for resolution of TAP and FFC (5 mg/mL). All chickens were injected intramuscularly with FFC (20 mg/kg), or TAP (20 mg/kg), or mixed FFC/TAP (at 2.5/10 or 5 /10 mg/kg) 30 min prior to intramuscular administration of 1 × 10^3^ CFU *P. multocida* (101-035) (1 mL volume). The clinical signs were observed every 6 h until the 3rd day.

### Uptake of TAP and FFC

The method used to determine the TAP uptake was following the assay of CAP uptake in a previous report (Burns et al., 1985). *P. multocida* (101-035) was grown to mid-log phase (~1 × 10^9^ CFU/mL) in BHI, washed once, and resuspended in 10 mL MHIIB containing 256 mg/L TAP alone or plus 8 mg/L of FFC. Subsequently, bacteria were incubated with shaking at 37°C, and 500 μL was harvested at 0, 15, 30, 45, and 60 min. Bacterial populations were counted at 0 and 60 min. The TAP and FFC concentration in supernatant in each sample was determined by high performance liquid chromatography (HPLC) as previously described (Burns et al., 1985).

### Cell permeability assay

The bacterial outer membrane permeability was measured using 1-*N*-phenylnaphthylamine (NPN) as a probe according to previous reports (Helander and Mattila-Sandholm, 2000; Ejim et al., 2011). NPN fluoresces strongly in phospholipid environments. Briefly, 96 well plates (black with clear bottom) were used and each wells contained 100 μL of *P. multocida* (101-035) suspension in 5 mM HEPES buffer (pH 7.2), 50 μL of 40 mM NPN and FFC at indicated concentration. A plate reader (Perkin-Elmer) was used with filters of 355 nm for excitation and 460 nm for emission and read every 10 min for 60 min at 25°C. Results were shown as relative fluorescent units calibrated to fluorescence in the absence of NPN.

### Uptake of OTC

The OTC uptake was evaluated via monitoring the fluorescence enhancement of oxytetracycline according to previous reports (Dockter et al., 1978; Ejim et al., 2011). *P. aeruginosa* (103-3430) grown in 10 mL TSB to OD_600_ = 0.8 were harvested at 2000 g for 10 min and then resuspended in 2.5 mL of 10 mM HEPES pH7.2. 100 μL of drug including 256 mM OTC and FFC at indicated concentrations was pipetted into wells (black with clear bottom). Bacterial suspension was added at 100 μL/well and the fluorescence read every 5 min for 60 min at 25°C. A plate reader (Perkin-Elmer) with filters of 405 nm for excitation and 535 nm for emission was employed. Results were performed as relative fluorescent units (RFU) corrected for fluorescence in the absence of bacteria suspension.

### Intracellular accumulation assay

The effect of FFC on the efflux pump was evaluated by ethidium bromide (EtBr) accumulation using semi-automated fluorometric method as described in previous reports (Viveiros et al., 2008). *P. multocida* (101-035) was grown in BHI at 37°C to OD_600_ of 0.8, washed once and resuspended in PBS (pH 7.4) containing 0.4% glucose to OD_600_ of 0.4. Aliquots of 95 μL bacterial suspension were transfer to 0.2-mL microtubes and EtBr was added at a final concentration of 2 mg/L. Rotor-Gene 3000 (Corbett Research) was employed to measure the fluorescence using 530 nm (excitation) and 585 nm (detection) wavelengths. Arbitrary units of fluorescence emitted by EtBr were acquired every minute for 30 min at 37°C.

### Multistep resistance by serial passage

The multistep resistance was assessed from two clinical *P. multocida* isolates by serial passage in exposure to FFC, TAP, or FFC/TAP combination. 50 mL of 1 × 10^8^ CFU/mL *P. multocida* were added to 10 mL MHIIB and incubated with sub-inhibitory concentrations of FFC, TAP, or FFC/TAP (see Table S2 for concentrations). The cultures were incubated at 37°C overnight and then were subcultured to fresh medium with antibiotics daily for 12 days. MIC was determined after every 3 passages using the microdilution method as described in Susceptibility testing.

### Statistical analysis

The bacterial populations in killing rate experiment were analyzed by 1-way Analysis of variance (ANOVA) and Tukey's test for multiple comparisons. Student's *t*-test was performed on the TAP uptake data. *P* values of < 0.05 were considered to be statistically significant. The software GraphPad Prism 6.0 was used for statistical analysis.

## Results

### Combination of FFC and TAP against bacterial pathogens *in vitro*

**Figure 1:**
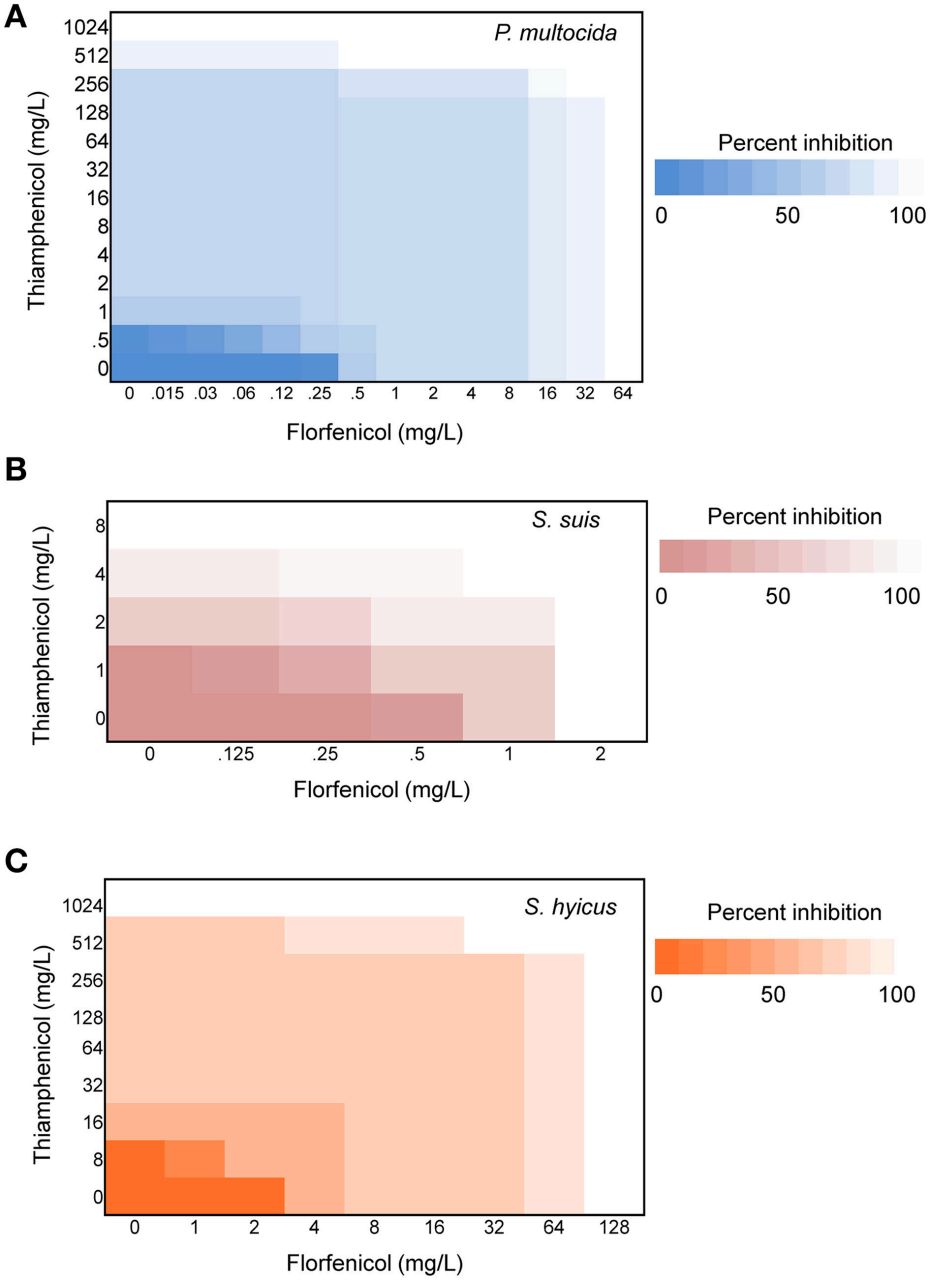
**Synergy of florfenicol and thiamphenicol**. Checkerboard analyses showing the percentage inhibition on the combined effect of florfenicol and thiamphenicol against **(A)** *P. multocida*, **(B)** *S. suis*, and **(C)** *S. hyicus*. The magnitude of inhibition is shown as a heat plot.

**Figure 2:**
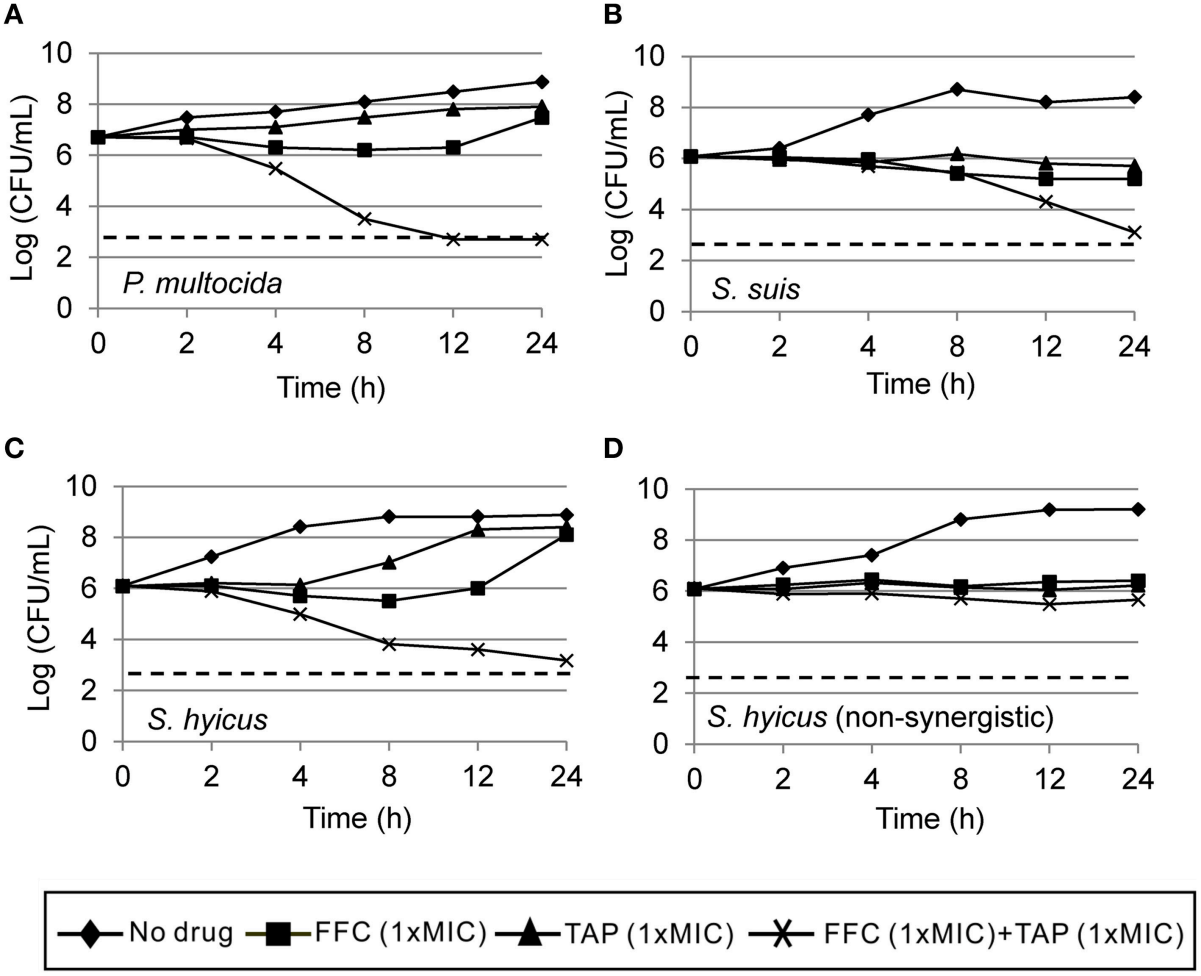
**Time-kill curves of combinations of florfenicol (FFC) and thiamphenicol (TAP) against (A) *P. multocida* (101-035), (B) *S. suis* (CY13005-1L), (C) *S. hyicus* (CM134-2), and (D) a non-synergistic strain, *S. hyicus* (CM144-1C) which serves as a negative control**. Refer to Table 1 for strain MIC data. Each point represents the mean of duplicate determinations. The detection limit (dotted line) was 2.7 log_10_ CFU/mL.

Initial assessment of antibacterial effect by microdilution checkerboard assay showed that the FFC MIC could be reduced to 1/16 MIC (*S. hyicus*) and 1/32 MIC (*P. multocida*) in combination with TAP at 1/2 MIC even though FICIs of all test strains are larger than 0.5 (Table 1). Moreover, the combination reached FICI values of < 0.75 in 70% of *P. multocida*, 62% of *S. suis*, and 50% of *S. hyicus* isolates tested, which could be consider as strong additive effects if assessed according to the European Committee for Antimicrobial Susceptibility Testing criteria (EUCAST, 2000) for synergy (Table 1). The antibacterial activity against bacteria species showing ≥50% additive effect were demonstrated as heat plots in Figure 1, which showed concentration ranges and their percentage inhibitions on tested strains listed in Table 1. Thirteen strains showing FICI ≤ 0.625 [one *S. hyicus*, two *S. suis* and ten *P. multocida* (Table 1)] were further assessed with the time-kill study to validate if they actually exhibited synergistic effects. At 24 h, all tested strains exposed to FFC/TAP combination (1 × MIC) decreased more than 2-log in CFU/mL compared to FFC or TAP alone (1 × MIC), suggesting the existence of synergy. The characteristic results for representative *S. hyicus, S. suis*, and *P. multocida* isolates are shown in Figure 2. In contrast, the combination was ineffective against *S. hyicus* (CM144-1C) (FICI = 2) and the time-kill assay showed corresponding result which was served as a negative control (Figure 2D). Furthermore, bactericidal effects on *P. multocida, S. suis*, and *S. hyicus* strains were observed when exposed to the combination (Figures 2A–C).

### Combination of FFC and TAP against *P. multocida in vivo*

**Figure 3:**
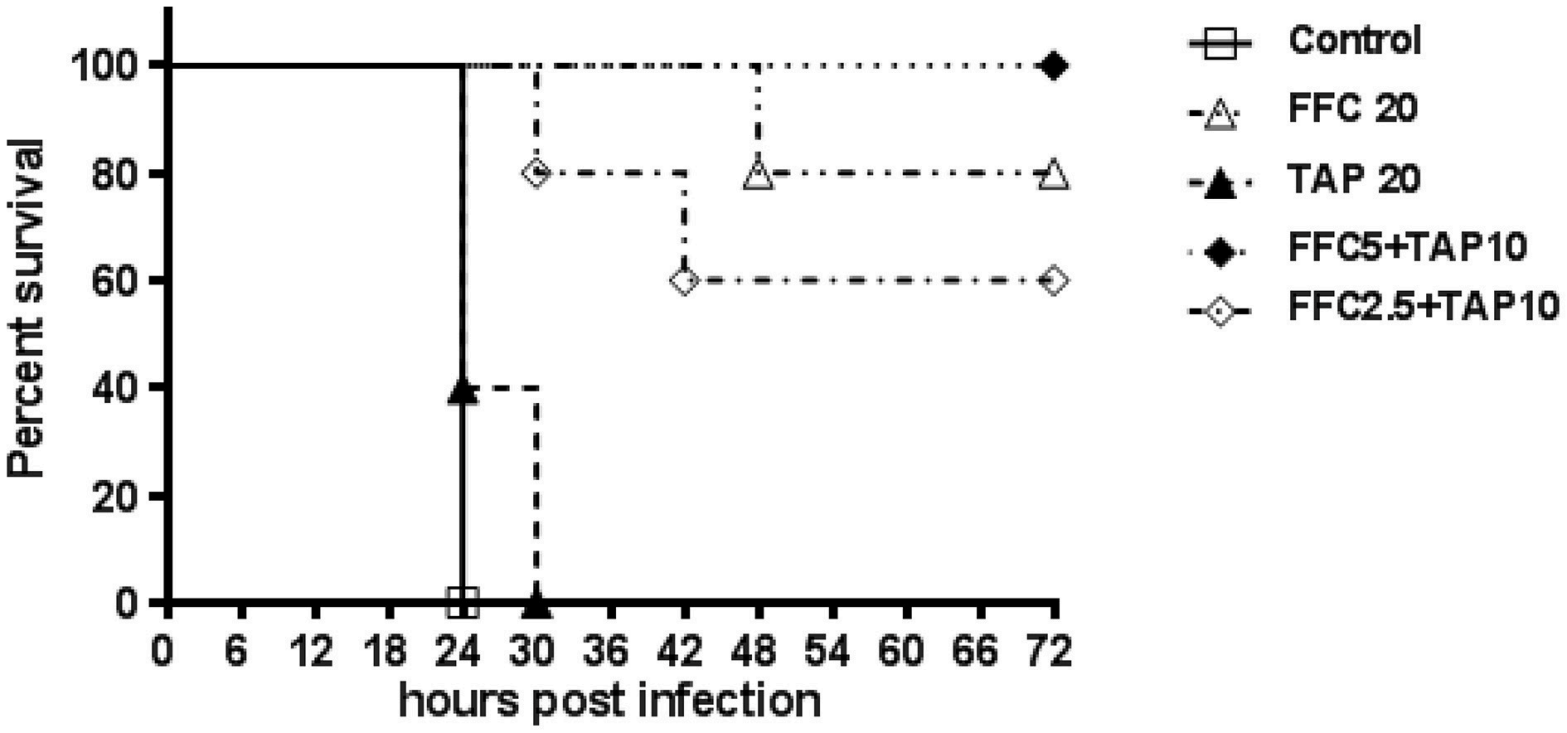
**Florfenicol (FFC)/thiamphenicol (TAP) combination protects broiler chickens from *P. multocida* infection**. Five groups of five broiler chickens challenged with 10^3^ CFU of *P. multocida* (101-035) and the mortality was recorded every 6 h until 72 h post infection.

The tested *P. multocida* strain 101-035 was originally isolated from a diseased goose. A fowl cholera model in broiler chickens by intramuscular injection of *P. multocida* was employed to assess the synergy *in vivo*. The challenged dose (>LD_50_) resulted in 100% death of chicken within 3 days. Clinical signs including anorexia and loss of activity were observed within 24 h post-infection for the control group and some medicated chickens. After 24 h, chickens began to die but the survived chickens could recover by 72 h post-infection. Subepicardial hemorrhages and multiple necrotic spots in liver were observed in the dead chickens. Judged by the survival rate at 72 h after infection, the combined dosage of FFC/TAP could be reduced to as low as 5 mg/kg/+10 mg/kg (1/4 FFC +1/2 TAP recommended dose) but still maintain 100% protection of chicken from death, indicating a strong synergy in the protection of *P. multocida* infection (Figure 3).

### FFC as an initiating sensitizer and modulator of antibiotic uptake

**Figure 4:**
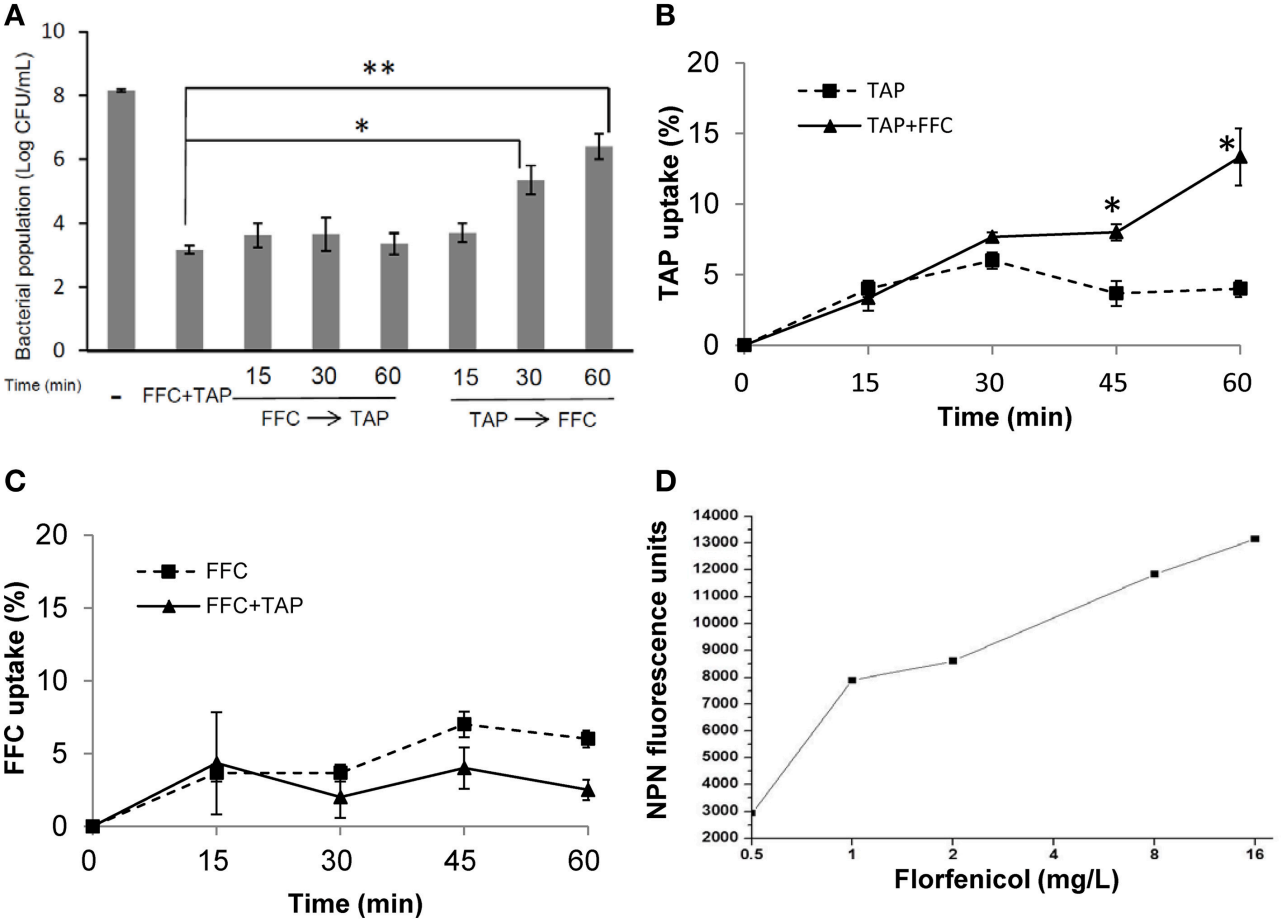
**Facilitated uptake of thiamphenicol (TAP) by florfenicol (FFC). (A)** Killing rates of florfenicol (FFC) and thiamphenicol (TAP) in different mixing times against *P. multocida*. Time indicates minutes FFC was added prior to (FFC → TAP) or after (TAP → FFC) TAP addition. (-) represented the control culture without addition of antibiotics. Bacteria populations were counted at 24 h and compared using one way ANOVA and Tukey's test for multiple comparisons. *P* values of less than 0.05 were considered significant. Statistical significance is presented as ^**^P < 0.01, or ^*^P < 0.05. **(B)** Uptake of TAP (depletion in medium) presented as a percentage of the initial concentration: 256 mg/L on *P. multocida*. The concentration of FFC was 8 mg/L. The data shown are the means ± SE of three independent experiments and were analyzed using Student's *t*-test. (^*^P < 0.05). **(C)** Uptake of FFC presented as a percentage of the initial concentration: 8 mg/L on *P. multocida*. The concentration of TAP was 256 mg/L. **(D)** Permeability of *P. multocida* was evaluated by measuring the 1-*N*-phenylnaphthylamine (NPN) at 10 min after FFC treatment at indicated concentration. The data shown are the average of triplicate experiments. The deviations between triplicate data point were < 5%.

To address the action mode of FFC/TAP combination, the antimicrobial activity at which the two drugs were added in the culture of *P. multocida* (101-035) at different order and time points were assessed. The bacterial population (CFU) was recorded at 8 h (Figure 4A). 1 × MIC of FFC added at 15, 30, and 60 min prior to the addition of 1 × MIC of TAP exerted the same antimicrobial activity as simultaneous addition of FFC and TAP. In contrast, TAP added first at 30 and 60 min prior to the addition of FFC showed reduced antimicrobial activity suggesting that FFC contributes to the initial and critical effect in the combination.

To further assess whether FFC promotes the entry of TAP across cell membrane, TAP concentration in the culture supernatants with or without FFC was quantified by HPLC. The bacterial populations remained comparable between TAP and TAP/FFC treatment over the 60-min incubation period. The uptake of TAP was significantly increased by 3 folds (from 4 to 13%) at 60 min when FFC (8 mg/L, final concentration) was present in the culture (Figure 4B). On the other hand, there was no significant difference in the uptake of FFC in the absence or presence of TAP (Figure 4C). To further assess whether FFC affect the outer membrane permeability, 1-*N*-phenylnapthylamine was employed as a fluorescent probe and found that FFC increased the membrane permeability of *P. multocida* (Figure 4D).

### Combination of FFC and OTC showed enhanced antibacterial activity against *P. aeruginosa*

**Figure 5:**
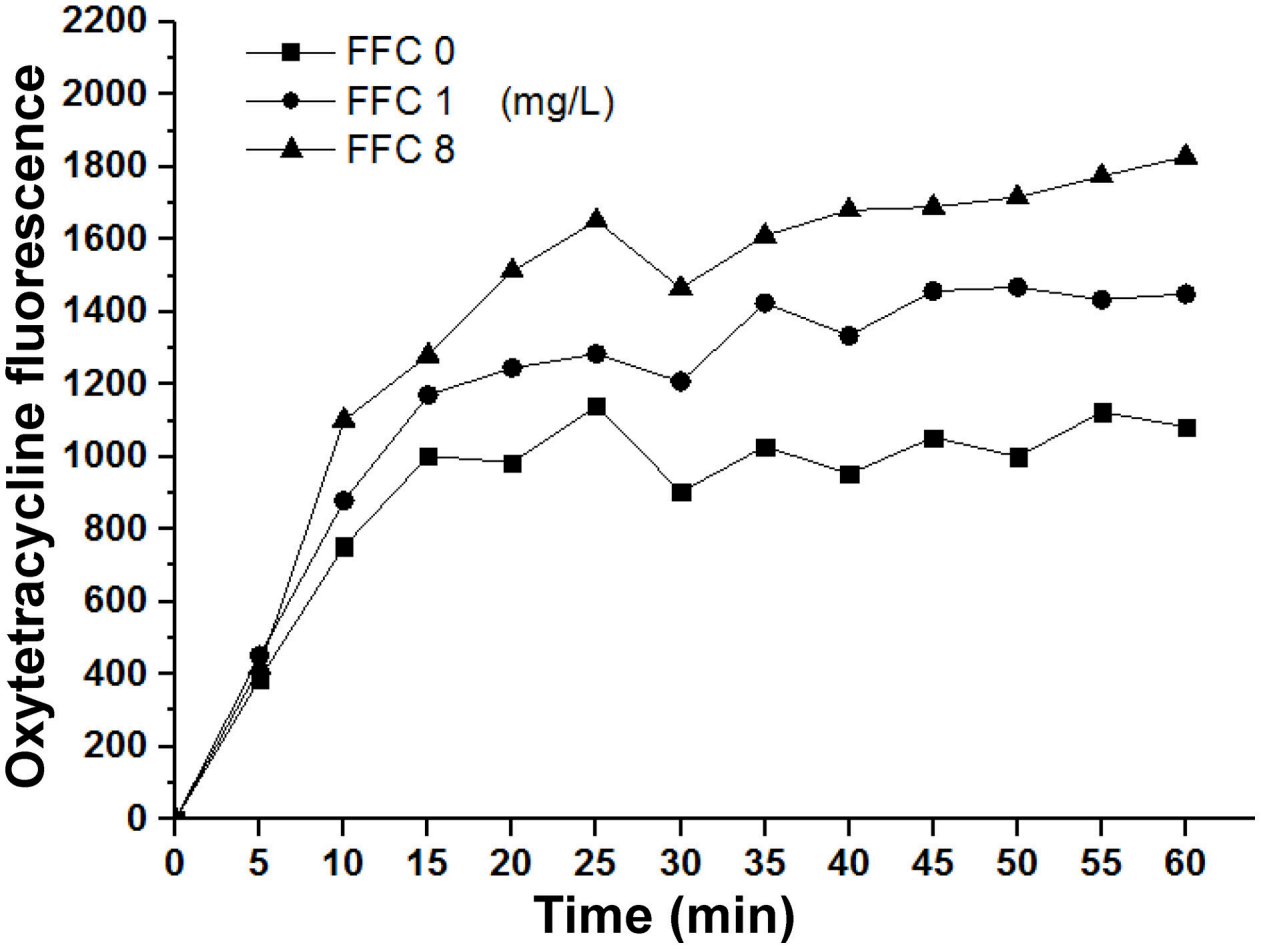
**Florfenicol (FFC) enhances the uptake of oxytetracycline in *P. aeruginosa***. Concentration of oxytetracycline was 128 mg/L, and FFC concentration was 0, 1, and 8 mg/L. Averages of triplicate experiments are shown.

To elucidate whether the enhancement of antibiotic entrance by FFC was specific to amphenicols, we further studied the uptake of OTC in the presence of sub-MIC level of FFC in *P. aeruginosa* (103-3430) via monitoring of the fluorescence enhancement of OTC in the cell. A dose-dependent increase of OTC by FFC was observed (Figure 5). The results correspond well to the checkerboard assay, FFC in combination with oxytetracycline (OTC) exhibited synergistic effect (FICI ≤ 0.5) against 4/9 (44%) of *P. aeruginosa in vitro* (Table 2).

**Table 2:** ***In vitro* inhibitory activity of florfenicol (FFC) and oxytetracycline (OTC) alone and in combination against *P*. *aeruginosa***.

**Strains**	**host**	**MIC (mg/L) of antibiotics alone**		**MIC (mg/L) in combination**	**Fold MIC in combination**		**FICI**
		**FFC**	**OTC**	**FFC + OTC**	**FFC**	**OTC**	
ATCC27853		256	32	32 + 16	1/8	1/2	0.625
103-3430	Rabbit	128	32	32 + 8	1/4	1/4	0.500
103-3713	Rabbit	256	32	64 + 8	1/4	1/4	0.500
27825	Rabbit	256	32	64 + 8	1/4	1/4	0.500
103-303	Rabbit	256	64	64 + 16	1/4	1/4	0.500
103-9432	Dog	512	64	128 + 32	1/4	1/2	0.75
103-1597	Dog	128	64	32 + 32	1/4	1/2	0.75
103-0044	Rabbit	256	1024	64 + 512	1/4	1/2	0.75
103-3190	Dog	512	64	256 + 32	1/2	1/2	1

### The effect of FFC on the efflux system of *P. multocida*

**Figure 6:**
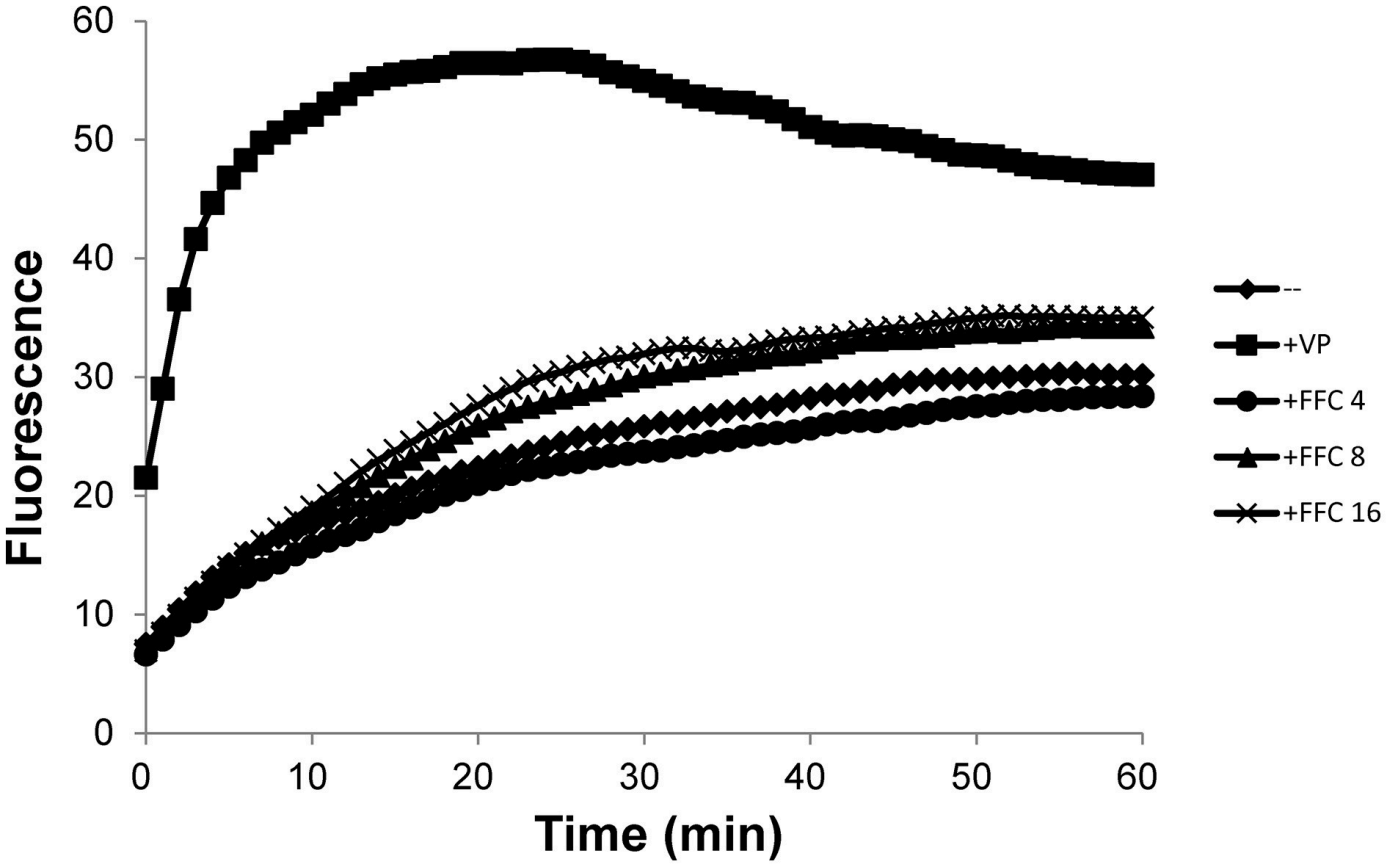
**Accumulation of ethidium bromide by *P. multocida* in the addition of increasing concentrations (mg/L) of florfenicol (FFC) at 37°C**. Verapamil (VP), a well-known efflux pump inhibitor, was added at 400 mg/L (1/2 MIC). The experiment was repeated three times with similar results.

To investigate possible role of efflux systems in FFC modulation, the semi-automated fluorometric method was used. In contrast to the efflux inhibitor, verapamil, which at 1/2 MIC significantly increased accumulation of EtBr in *P. multocida* (101-035); FFC at 4, 8, and 16 mg/L only resulted in insignificant increase of EtBr accumulation inside the cells (Figure 6). A marginal accumulation of EtBr was observed and might be attributed to the increase of membrane permeability or the inhibition of a minor efflux pump. To further elucidate whether the expressions of efflux pumps are affected by FFC, the transcriptome of *P. multocida* (101-035) growing in the presence and absence of FFC at sub-MIC levels were compared using Illumina RNA-seq. Based on the general criterion of gene expression (fold change ≤ 2 or > 2 at *P* values < 0.02); out of the 2190 open reading frames (ORFs) predicted in the *P. multocida* genome, the sub-MIC FFC treatment caused 74 (3.3%) ORFs upregulated and 65 (3.0%) ORFs downregulated (Table S3). However, no efflux pump-related gene was significantly up or down regulated in the sub-MIC FFC treatment (Table S3).

### Assessment of FFC/TAP resistance induction in sub-inhibitory concentrations by serial passage of *P. multocida*

**Figure 7:**
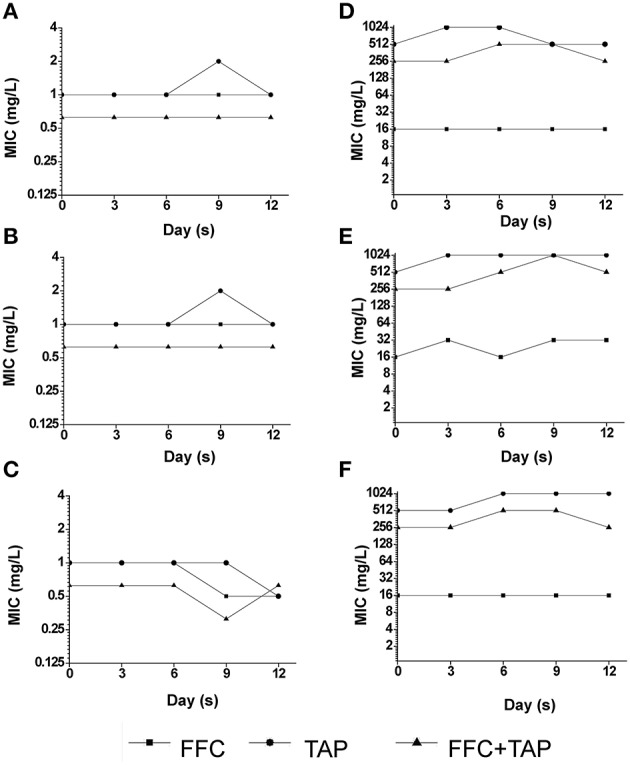
**Development of resistance after serial exposure to florfenicol (FFC), thiamphenicol (TAP) and FFC+TAP**. Serial passage of *P. multocida* (0425) **(A–C)** and (101-035) **(D–F)** exposed to sub-MIC levels of florfenicol (FFC) **(A,D)**, thiamphenicol (TAP) **(B,E)**, and FFC+TAP **(C,F)**. MIC of each point was determined at least in triplicates. The experiment was repeated three times with similar results.

Recent studies revealed that exposure to sub-MIC antibiotic has an important role on the evolution of antibiotic resistance (Baquero et al., 1998; Andersson and Hughes, 2014). To address whether the combination of FFC and TAP in sub-inhibitory concentration may accelerate the development of resistance in *P. multocida*, the emergence of multistep resistance were evaluated by serial passage of bacteria every day for 12 days in sub-inhibitory concentration level (See Table S2 for concentrations). After 12 days of passage, the MIC of FFC/TAP combination in susceptible strain was not changed when grown with single antibiotic or combination (Figures 7A–C). Interestingly, unlike exposure to single antibiotic, exposure to FFC/TAP combination resulted in 2-fold decrease in FFC and TAP MIC (Figure 7C). Likewise, the MIC of FFC/TAP combination in resistant strain was not increased when grown in either FFC or the FFC/TAP combination (Figures 7D,F). However, the MICs of FFC, TAP, and FFC/TAP combination elevated 2 folds when exposed to TAP for 12 days (Figure 7E).

## Discussion

Florfenicol is mainly administrated to combat bovine respiratory infections, it showed a broad spectrum of activity against Gram-negative bacteria such as *E. coli, K. pneumoniae, Proteus valgaris*, and *P. multocida* and also some Gram-positive bacteria such as *S. aureus* (Neu and Fu, 1980) and *S. suis* (Ferreira et al., 2007). The current results showed general agreement with the published data in antibacterial spectrum. Although, FFC was effective against *P. multocida, S. suis*, and *S. hyicus*, its analog TAP was shown to be much less effective than FFC (Table 1) and virtually not effective in practice. Therefore, the significant dose reduction by 75% in FFC and 50% in TAP and still maintained the same antimicrobial effectiveness both *in vitro* and *in vivo* has made the combination a notable case worth pursuing.

The synergism between the same class of antibiotics seemed unforeseeable. In fact, it was first observed as an incidental finding in a study of heat stability of antibiotics. It was then assessed by multiple microbiological evaluations that are accepted as standard measures for identification of synergistic activities. The checkerboard method was first applied to assess the interaction of FFC and TAP. In a 2-fold dilution process, although the FFC MIC of certain strains could be reduced to 1/32 of the original, the MIC reduction for TAP could only reach 1/2, which rendered all FICIs to be above 0.5 (additivity by definition). In order to elucidate whether the combination reaches synergistic effect (FICI ≤ 0.5), the giant checkerboard (Horrevorts et al., 1987; Hsieh et al., 1993) was then employed for further dilution of TAP to between 1/2 MIC and 1/4 MIC. The results revealed that the combination actually exerted a strong synergistic effect (FICI ≤ 0.5) on 16/21 (77%) of the strains whose FICI was originally between 0.5 and 0.625 (Table S4). The synergism was also confirmed by another *in vitro* method, time-kill study. The methods employed to assess the synergism including checkerboard and time-kill assay have been compared in several reports (Weinstein et al., 1975; Norden et al., 1979; Bayer and Morrison, 1984; White et al., 1996; Rose et al., 2013). Certain studies revealed that the time-kill assay is more reliable in the prediction of *in vivo* synergism (Bayer and Morrison, 1984; Chadwick et al., 1986) and exhibited synergy with higher frequency than did the checkerboard (Norden et al., 1979; Bayer and Morrison, 1984; White et al., 1996). In current study, we also observed the synergism with higher frequency in the time kill assay.

Interestingly, Gram-positive species, *S. hyicus* and *S. suis*, showed higher frequency of additive effect and synergistic effects in some strains in the initial checkerboard screening, suggesting that this combination mainly affect Gram-positive bacteria. Gram-negative bacteria are known to contain a surrounding outer membrane severing as a barrier to nonpolar compound or certain antibiotics (Nikaido and Nakae, 1979; Nikaido and Vaara, 1985). Therefore, it is possible that the FFC/TAP combination falls in this category and was obstructed by the outer membrane and thereby exhibited less synergistic effect. Nevertheless, among the five Gram-negative species tested, *P. multocida* showed high percentage of susceptibility to FFC/TAP interaction. This might be explained by the high permeability of outer membrane for nonpolar compounds (Watt et al., 2003; Ellison and Champlin, 2007) in this species, which render it more susceptible to distinct hydrophobic antimicrobial agents despite that the cell envelope ultrastructure of *P. multocida* is characteristically a Gram-negative bacteria (Hart and Champlin, 1988). Results from Figures 4A,B showed that the effect of FFC preceded TAP (not in reversed order) in the exertion of synergistic activity, likely through the increased membrane permeability (indicated by NPN) that resulted in significantly increased (>3 folds) intracellular TAP concentration (Figure 4D). Therefore, it is feasible to assume that FFC/TAP combination works in an orderly manner, in which FFC acted as an initiating sensitizer to enhance the entrance of cognate antibiotics (such as TAP) through the bacterial cytoplasm. The role of FFC as an initiator (enhancer) was further demonstrated in its combination to other class of antibiotic OTC. Notably, FFC by itself is not an effective drug against *P. aeruginosa*, most strains of *P. aeruginosa* were intrinsically resistant to CAP due to very low permeability of their outer membrane and the efforts of efflux pumps (Angus et al., 1982; Li et al., 2015). However, FFC in combination with OTC showed synergistc effect against *P. aeruginosa* due to enhancement of OTC uptake (Table 2, Figure 5). Our preliminary investigation also revaled that sub-MIC FFC is able to cripple *P. aeruginosa* in swimming motility assay (data not shown), suggesting that FFC might affect the flagellar function via abolishing the proton motive force to triger the uptake of OTC (Paul et al., 2008). Therefore, the latter result may represent a different type of combinational antibacterial effect. Nevertheless, both results supported that FFC acts as an initiating modulator of membrane permeability that allows increased uptake of multiple antibiotics by susceptible Gram-negative bacteria.

In addition to the permeability barrier of outer membrane, the multidrug pumps (efflux pumps) usually contribute the antibiotic resistance in synergy with outer membrane (Li et al., 2015). Thus, the accumulation of EtBr was employed to address whether FFC act as an efflux pump inhibitor and RNA-seq was used to evaluate the expression of efflux pump-related genes. Our data showed slight accumulation of EtBr as FFC concentration is increased may reflect minor inhibitory effect of efflux pump such as resistance-nodulation-division (RND)-type transporter. However, transcriptomic analysis performed in the presence or absence of sub-MIC FFC revealed that the expression of efflux pump-related genes was not significantly altered. So far, whether FFC serves as an efflux pump inhibitor is not confirmed and requires more investigation. This study raises an interesting observation that FFC acts as a modulator not only promote uptake of OTC, but also TAP even though they are analogs. Further investigations are required to address the mechanism, including the reverse transcription-PCR to validate the observed gene expression. In addition, Blickwede et al. demonstrated that sub-MIC of FFC results in a distinct thickening of the staphylococcal cell wall and might cause cellular disruption due to a compression of the protoplast (Blickwede et al., 2004). Thus, for Gram-positive bacteria, another plausible hypothesis to support the synergistic effect could be that the FFC at sub-MIC concentration condenses the bacterial protoplast leading to higher intracellular TAP concentration. Further, studies investigating whether FFC predisposition also synergizes with other classes of antibiotics against *Staphylococcus* spp. or other Gram-positive bacteria may shed more light on this hypothesis.

*P. multocida* is the causative agent of primary infections in the domestic animals as well as opportunistic infections in humans (Wilson and Ho, 2013). Intramuscular injection with *P. multocida* to cause fowl cholera was demonstrated as a reliable and reproducible model (Sarközy et al., 2002). In the chicken model, a FFC-resistant strain but showing significant susceptibility to the FFC/TAP combination *in vitro* was used. The results indicated that the antimicrobial activity was dose-dependent after FFC/TAP co-administration, while 80% of challenged-chicken survived at the recommended FFC dose (20 mg/kg), the co-administration at lower dosage was able to protect 100% chickens, suggesting better efficacy. It is also worth noting that the combination restores a drug (TAP) that is not very effective when used alone. Interestingly, the ratio of the dosage combination was proportional to their *in vitro* MIC results. Whether or not this ratio carries any mechanistic importance remains to be elucidated, but this combination significantly reduced the amounts of drugs administered, and it is plausible that a favorable pharmacokinetic behavior leading to less unwanted tissue residues and side effects are expected and the cost for treatment could be largely reduced with this combinational therapy.

In summary, this study is the first to report FFC as a possible synergistic antibacterial modulator to multiple antibiotic classes and is effective against both Gram-positive and Gram-negative bacteria depending on the antibiotics in combination. The antibacterial spectrum, *in vitro* bactericidal activities, *in vivo* effectiveness, and mechanisms of action were systematically investigated using combination with another amphenicol TAP as an example. When used in sub-MIC levels (usually 1/4 of its original MIC), FFC presumably exhibited synergistic antibacterial activity through alteration of bacterial membrane permeability and subsequent increase of intracellular concentration of the antibiotics used for combination. The efflux pumps commonly responsible for reduced therapeutic effect and the development of antibacterial resistance were yet to be proven in the mechanism based on EtBr accumulation assay and RNA sequencing. The current discovery provided abundant evidence to support further development of drug combinations involving FFC as an initiating modulator.

## Author contributions

CW and CC conceived and designed the study. CW, JS, SC, and CC participated in the experiment. CW and CC analyzed the data and drafted the manuscript. All authors read and approved the final manuscript.

### Conflict of interest statement

The authors declare that the research was conducted in the absence of any commercial or financial relationships that could be construed as a potential conflict of interest.
