# Supplementary material for: Florfenicol As a Modulator Enhancing Antimicrobial Activity: Example Using Combination with Thiamphenicol against Pasteurella multocida
Source: Front Microbiol. 2016 Mar 30;7:389. doi: 10.3389/fmicb.2016.00389 (PMC4811925; doi:10.3389/fmicb.2016.00389)
Supplement: Supplementary file 1 [file Table1.PDF]

Supplementary Material

Florfenicol as a modulator enhancing antimicrobial activity: example using combination with thiamphenicol against *Pasteurella multocida*

Chia-Fong Wei, Jui-Hung Shien, Shao-Kuang Chang, and Chi-Chung Chou*

* Corresponding author

E- mail address: ccchou@nchu.edu.tw (C.C. Chou)

1 Supplementary Methods

1.1. Sample preparation for RNA-seq and sequencing procedures

P. multocida strain 101-035 was grown overnight in BHI medium at 37 °C, refreshed in the same medium to an OD_{600} of 0.8, washed once and resuspended in MHIIB containing FFC at sub-lethal concentrations (8 mg/L). Subsequently, total RNA was extracted by Trizol® Reagent (Invitrogen) according to the instruction manual. The RNA quality was assessed by a Bioanalyzer 2100 (Agilent Technology) with RNA 6000 labchip kit (Agilent Technologies). Construction of cDNA Libraries was carried out using Agilent's SureSelect Strand Specific RNA Library Preparation Kit for 75SE bp sequencing on Solexa platform according to the manufacture’s protocol (Illumina). Raw sequences were processed from the Illumina Pipeline software bcl2fastq v2.0.

1.2. RNA-seq Analysis

Raw sequences were initially filtered using NGS QC Toolkit to obtain qualified

reads. Trimmomatics was employed to trim or remove the reads according to the quality score. Quality-filtered reads were analyzed using TopHat/Cufflinks for gene expression estimation. FPKM (fragments per kilobase of transcript per million mapped reads) was calculated as gene expression level. For differential expression analysis, CummeRbund was employed to perform statistical analyses of gene expression profiles. The reference genome (*Pasteurella multocida* subsp. *multocida* str. HB03) and gene annotations were retrieved from Ensembl database.

2. Supplementary Tables

Table S1. *In vitro* inhibitory activity of florfenicol (FFC) and thiamphenicol (TAP) alone and in combination against clinical pathogens**.**

Strains	host	MIC (mg/L) of antibiotics alone		MIC (mg/L) in combination	Fold MIC in combination		FICI
		FFC	TAP	FFC+TAP	FFC	TAP	
Standard strains							
E. coli ATCC25922		8	64	2+32	1/4	1/2	0.75
S. Typhimurium ATCC19585		8	32	2+16	1/4	1/2	0.75
*P. aeruginosa*ATCC27853		256	256	128+128	1/2	1/2	1
Clinical isolates							
Escherichia coli							
CCC01	Ch	8	64	2+32	1/4	1/2	0.75
CCC07	G	16	128	4+64	1/4	1/2	0.75
CM139-1	Ch	8	128	2+64	1/4	1/2	0.75
CP11043-1	T	1024	1024	256+512	1/4	1/2	0.75
0705-2	C	8	128	2+64	1/4	1/2	0.75
0120-1-1	D	1024	1024	256+512	1/4	1/2	0.75
CCC03	Ch	256	1024	128+512	1/2	1/2	1
CCC04	Ch	256	1024	128+512	1/2	1/2	1
CCC34	Ca	8	128	4+64	1/2	1/2	1
CCC35	Ca	8	128	4+64	1/2	1/2	1
CM78-1	Ch	1024	1024	512+512	1/2	1/2	1
CP11038	P	8	128	4+64	1/2	1/2	1
CS12007-1	Pig	8	1024	4+512	1/2	1/2	1
CD12057-2	Pig	8	1024	4+512	1/2	1/2	1
CD12058-2	Pig	2	1024	1+512	1/2	1/2	1
CD12013-1	Pig	256	512	128+256	1/2	1/2	1
CS12010-3	Pig	256	2048	128+1024	1/2	1/2	1
0729-1	Pig	8	128	4+64	1/2	1/2	1
0222-2	Pig	512	1024	256+512	1/2	1/2	1
0512-6	D	2	1024	1+512	1/2	1/2	1
CCC33	Ca	8	128	8+128	1	1	2
CJ12002-2	Pig	128	1024	128+1024	1	1	2
1003-1-2	C	512	1024	512+1024	1	1	2
0701-1	D	256	1024	256+1024	1	1	2
1006-3	D	256	1024	256+512	1	1	2

Salmonella entrica							
Typhimurium CCCSZ28	Pig	8	64	2+32	1/4	1/2	0.75
Typhimurium CCCSZ27	Pig	8	64	4+32	1/2	1/2	1
Typhimurium CCCSZ30	Pig	8	64	4+32	1/2	1/2	1
Typhimurium CM49-2	Pig	512	1024	256+512	1/2	1/2	1
Hader CM148	Pig	8	128	4+64	1/2	1/2	1
Brunei CCCSZ38	Pig	2	16	1+8	1/2	1/2	1
Pullorum CCC13	Ch	4	16	2+8	1/2	1/2	1
Albany B14	T	128	1024	64+512	1/2	1/2	1
Derby CJ12002-1	Pig	1024	1024	512+512	1/2	1/2	1
Choleraesuis CM148	Pig	1024	1024	512+512	1/2	1/2	1
Typhimurium G060	G	8	1024	8+1024	1	1	2
Typhimurium D150	Du	512	1024	512+1024	1	1	2
Typhimurium CCCSZ29	Pig	512	1024	512+1024	1	1	2
Hader CCCSZ26	Pig	8	64	8+64	1	1	2
Choleraesuis CCCSZ33	Pig	8	1024	8+1024	1	1	2
Albany B02	B	128	1024	128+1024	1	1	2
Schwarzengrund L21	T	512	1024	512+1024	1	1	2
Livingstone CCCSZ37	Pig	512	1024	512+1024	1	1	2
Pseudomonas aeruginosa							
103-0044	R	256	256	64+128	1/4	1/2	0.75
103-3430	R	128	128	32+64	1/4	1/2	0.75
103-1597	D	128	128	32+64	1/4	1/2	0.75
103-9432	D	512	1024	256+512	1/2	1/2	1
103-3713	R	256	256	128+128	1/2	1/2	1
27825	Tt	256	256	128+128	1/2	1/2	1
103-303	D	256	256	128+128	1/2	1/2	1
103-3190	D	512	2048	512+2048	1	1	2
Riemerella anatipestifer							
CCC26	Du	4	256	2+128	1/2	1/2	1
CCC27	Du	2	64	1+32	1/2	1/2	1
CCC28	Du	4	256	2+128	1/2	1/2	1

CCC29	Du	4	64	2+32	1/2	1/2	1

FICI, fractional inhibitory concentration index; FFC, florfenicol; TAP, thiamphenicol

Host: B, Bird; Ca, Cattle; C, Cat; Ch, Chicken; D, Dog; Du, Duck; G, Goose; R, Rabbit; Pig, Pig; P, Pigeon; T, Turkey; Tt, Turtle

Table S2. Concentrations (mg/L) of florfenicol, thiamphenicol and florfenicol/thiamphenicol combination used in multistep resistance assay.

P. multocida strains	FFC	TAP	FFC/TAP
0425	0.02	0.04	0.06
101-035	0.1	20	20.1

FFC, florfenicol; TAP, thiamphenicol

Table S3. Differential expression of ORFs in 4 h treatment of florfenicol (FFC) versus in no treatment (Control).

			FPKM		FFC/Control		Statistics	
Gene ID	Gene	Description	Control	FFC	Ratio	Log2 Ratio	*p* value	*q* value
PMCN03_0118	mglA	galactose/methyl galaxtoside transporter ATP-binding protein [Pasteurella multocida subsp. multocida st	29.4164	1176.37	39.99027753	5.32157739	0.02175	0.597743
PMCN03_1974	rpl10	50S ribosomal protein L10 [Pasteurella multocida subsp. multocida str. Pm70]	1246.87	44648.7	35.8086248	5.16223521	0.00575	0.553183
PMCN03_1975	rpl12	50S ribosomal protein L7/L12 [Pasteurella multocida subsp. multocida str. Pm70]	2494.07	86636.2	34.73687587	5.1183961	0.005	0.553183
PMCN03_0117	mglC	galactoside transport system permease protein MglC [Pasteurella multocida 36950]	46.8909	1512.51	32.25593879	5.0114929	0.02695	0.608707
PMCN03_1971	rpl1	50S ribosomal protein L1 [Pasteurella multocida subsp. multocida str. Pm70]	461.548	10340.5	22.40395365	4.48568144	0.02455	0.608707
PMCN03_1668	-	transmembrane protein [Pasteurella multocida 36950]	34.6558	771.8	22.27044247	4.47705832	0.0022	0.53676
PMCN03_1970	rpl11	50S ribosomal protein L11 [Pasteurella multocida subsp. multocida str. Pm70]	882.258	19275.1	21.84746412	4.44939393	0.0256	0.608707
PMCN03_1924	corA	magnesium/nickel/cobalt transporter CorA [Pasteurella multocida subsp. multocida str. Pm70]	54.7572	1013.49	18.50879884	4.21013937	0.0038	0.53676
PMCN03_1597	rps3	30S ribosomal protein S3 [Pasteurella multocida subsp. multocida str. Pm70]	1084.24	19676.4	18.14764259	4.18171025	0.02195	0.597743
PMCN03_0130	metK	S-adenosylmethionine synthetase [Pasteurella multocida subsp. multocida str. Pm70]	25.8487	457.783	17.71009761	4.14650026	0.00625	0.553183
PMCN03_1901	rps16	30S ribosomal protein S16 [Pasteurella multocida subsp. multocida str. Pm70]	803.95	14079.3	17.51265626	4.13032602	0.00305	0.53676
PMCN03_1902	rimM	ribosome maturation factor RimM [Pasteurella multocida 36950]	326.318	5491.92	16.82996341	4.07296013	0.04675	0.681498
PMCN03_1598	rpl22	50S ribosomal protein L22 [Pasteurella multocida 36950]	2503.12	41071.1	16.40796286	4.03632423	0.0363	0.644325
PMCN03_1669	-	transmembrane protein [Pasteurella multocida 36950]	52.5846	817.589	15.54806921	3.95866353	0.0009	0.53676
PMCN03_0605	rpl35	50S ribosomal protein L35 [Pasteurella multocida subsp. multocida str. Pm70]	1638.46	25032.4	15.27800496	3.93338426	0.0053	0.553183
PMCN03_1904	rpl19	50S ribosomal protein L19 [Pasteurella multocida subsp. multocida str. Pm70]	377.968	5712.35	15.11331647	3.91774838	0.0044	0.5467
PMCN03_0568	codA	cytosine deaminase [Pasteurella multocida 36950]	26.3855	387.883	14.70061208	3.87780432	0.00755	0.584931
PMCN03_1002	-	putative protease [Pasteurella multocida 36950]	10.2205	143.901	14.07964385	3.81553894	0.0119	0.597743
PMCN03_1603	rpl3	50S ribosomal protein L3 [Pasteurella multocida subsp. multocida str. Pm70]	1400.29	19143.6	13.67116812	3.77306461	0.03155	0.608707
PMCN03_1600	rpl2	50S ribosomal protein L2 [Pasteurella multocida subsp. multocida str. Pm70]	1030.34	14012.5	13.59987965	3.76552198	0.0309	0.608707
PMCN03_1196	-	hemolysin activation/secretion protein-1 [Pasteurella multocida 36950]	66.1979	889.5	13.43698214	3.74813725	0.00135	0.53676
PMCN03_0658	msmB	protein MsmB [Pasteurella multocida subsp. multocida str. Pm70]	316.653	3817.71	12.05644665	3.59173286	0.00185	0.53676
PMCN03_1542	-	Trap-type c4-dicarboxylate transport system, small permease component [Pasteurella multocida 36950]	60.0562	699.368	11.64522564	3.54166669	0.0173	0.597743
PMCN03_1095	yqhD	alcohol dehydrogenase YqhD [Pasteurella multocida 36950]	64.4367	725.203	11.25450248	3.49243038	0.01215	0.597743
PMCN03_0784	hyaE	protein HyaE [Pasteurella multocida 36950]	14.9263	165.056	11.05806529	3.46702709	0.01725	0.597743
PMCN03_0067	fis	DNA-binding protein Fis [Pasteurella multocida subsp. multocida str. Pm70]	924.207	10019	10.84064501	3.43837869	0.00715	0.584931
PMCN03_0947	lldD	L-lactate dehydrogenase [Pasteurella multocida 36950]	8.93455	96.8323	10.8379605	3.43802139	0.0039	0.53676
PMCN03_0974	potD	spermidine/putrescine-binding periplasmic protein PotD1 [Pasteurella multocida 36950]	90.6618	961.569	10.60610974	3.40682368	0.01	0.597743
PMCN03_0300	-	drug/metabolite transporter [Pasteurella multocida 36950]	82.3887	854.627	10.37310942	3.37477651	0.01245	0.597743
PMCN03_1654	pabA	putative anthranilate synthase component II [Pasteurella multocida 36950]	12.4818	129.352	10.36324889	3.37340446	0.01465	0.597743
PMCN03_0888	ilvI	acetolactate synthase large subunit [Pasteurella multocida 36950]	30.7337	308.423	10.0353358	3.32701699	0.01505	0.597743
PMCN03_1159	alsT	putative transporter [Pasteurella multocida 36950]	17.6562	167.81	9.504310101	3.24858191	0.0264	0.608707
PMCN03_1786	murQ	N-acetylmuramic acid-6-phosphate etherase [Pasteurella multocida]	132.083	1230.42	9.315506159	3.21963416	0.01385	0.597743
PMCN03_0838	holC	DNA polymerase III subunit chi [Pasteurella multocida 36950]	220.158	2042.17	9.275929105	3.21349179	0.02465	0.608707
PMCN03_0844	grx	glutaredoxin [Pasteurella multocida subsp. multocida str. Pm70]	565.292	5189.51	9.180228979	3.19853014	0.02805	0.608707
PMCN03_0597	thrS	threonyl-tRNA synthetase [Pasteurella dagmatis]	71.4155	653.785	9.154665304	3.19450714	0.0113	0.597743
PMCN03_1397	brnQ	putative branched-chain amino acid carrier protein SSP1343 [Pasteurella multocida 36950]	40.6479	364.035	8.955813215	3.16282444	0.0253	0.608707
PMCN03_1765	tfoX	DNA transformation protein TfoX [Pasteurella multocida 36950]	17.3518	153.815	8.864498208	3.14803897	0.00765	0.584931
PMCN03_1585	rpl30	50S ribosomal protein L30 [Pasteurella multocida subsp. multocida str. Pm70]	1679.17	14412.1	8.5828713	3.10146037	0.03505	0.639384
PMCN03_1275	pilF	tetratricopeptide-like helical family protein [Pasteurella multocida 36950]	30.0661	257.953	8.579529769	3.10089858	0.00545	0.553183
PMCN03_2162	rpl34	50S ribosomal protein L34 [Pasteurella multocida subsp. multocida str. Pm70]	6614.78	55458.8	8.384073242	3.06765132	0.0284	0.608707
PMCN03_0720	-	neurotransmitter symporter [Pasteurella multocida 36950]	22.3355	185.539	8.306910524	3.05431201	0.0318	0.608707
PMCN03_0471	-	hypothetical protein Pmu_05380 [Pasteurella multocida 36950]	78.6538	638.517	8.118069311	3.02113666	0.0317	0.608707
PMCN03_0642	rpl25	50S ribosomal protein L25 [Pasteurella multocida subsp. multocida str. Pm70]	711.639	5719.05	8.036448255	3.00655804	0.03005	0.608707
PMCN03_1538	uxuA	mannonate dehydratase [Pasteurella multocida subsp. multocida str. 3480]	138.456	1112.38	8.034176923	3.00615023	0.01555	0.597743
PMCN03_1276	-	ribosomal RNA large subunit methyltransferase N [Pasteurella multocida subsp. multocida str. Pm70]	18.419	147.972	8.033660894	3.00605757	0.03715	0.645668
PMCN03_1642	yaaH	hypothetical protein PM1452 [Pasteurella multocida subsp. multocida str. Pm70]	80.2886	636.368	7.92600693	2.98659423	0.03195	0.608707
PMCN03_0302	obgE	GTPase ObgE [Pasteurella multocida subsp. multocida str. Pm70]	105.918	839.066	7.921845201	2.98583651	0.0199	0.597743
PMCN03_0085	-	membrane protein, aromatic hydrocarbon degradation family [Pasteurella multocida 36950]	106.068	826.396	7.791190557	2.9618438	0.02015	0.597743
PMCN03_1748	gabD	succinate-semialdehyde dehydrogenase [Pasteurella multocida 36950]	103.074	799.96	7.761026059	2.9562474	0.02055	0.597743
PMCN03_1923	-	hypothetical protein PM1314 [Pasteurella multocida subsp. multocida str. Pm70]	82.9874	633.36	7.632001967	2.93206154	0.03555	0.639384
PMCN03_1356	rpl32	50S ribosomal protein L32 [Pasteurella multocida subsp. multocida str. Pm70]	1305.47	9826.9	7.527480524	2.91216707	0.03875	0.647332
PMCN03_0936	-	hypothetical protein NT08PM_0338 [Pasteurella multocida subsp. multocida str. 3480]	619.24	4634.73	7.484545572	2.90391472	0.01355	0.597743
PMCN03_1273	gcpE	4-hydroxy-3-methylbut-2-en-1-yl diphosphate synthase [Pasteurella multocida 36950]	71.1422	522.623	7.346174282	2.87699312	0.0392	0.647332
PMCN03_0266	iscR	hypothetical protein PM0317 [Pasteurella multocida subsp. multocida str. Pm70]	191.45	1398.44	7.304465918	2.86877879	0.03895	0.647332
PMCN03_2068	pdxA	4-hydroxythreonine-4-phosphate dehydrogenase [Pasteurella multocida subsp. multocida str. Pm70]	78.2673	568.986	7.269779333	2.86191157	0.0402	0.649737
PMCN03_0048	abgT	hypothetical protein PM1104 [Pasteurella multocida subsp. multocida str. Pm70]	45.674	331.205	7.251499759	2.8582794	0.0389	0.647332
PMCN03_0959	dctP	hypothetical protein PM0275 [Pasteurella multocida subsp. multocida str. Pm70]	76.0312	514.559	6.767734825	2.75867304	0.044	0.675155
PMCN03_1837	-	hypothetical protein, partial [Pasteurella multocida]	148.677	1004.42	6.755718773	2.75610927	0.04515	0.677116
PMCN03_1313	srlB	glucitol/sorbitol-specific phosphotransferase enzyme IIA component [Pasteurella multocida 36950]	24.5662	164.048	6.677793065	2.73937139	0.01185	0.597743
PMCN03_1274	-	transmembrane protein [Pasteurella multocida 36950]	82.5435	539.478	6.535681186	2.70833761	0.04745	0.681498
PMCN03_0845	argAB	amino-acid acetyltransferase [Pasteurella multocida 36950]	10.4383	65.4575	6.270896602	2.64867173	0.01945	0.597743
PMCN03_1337	-	hypothetical protein Pmu_14010 [Pasteurella multocida 36950]	25.4071	157.473	6.1979919	2.63180087	0.0286	0.608707
PMCN03_1015	purD	phosphoribosylamine--glycine ligase [Pasteurella multocida 36950]	130.53	791.694	6.065226385	2.60056149	0.037	0.645668
PMCN03_1032	-	hypothetical protein PMCN06_1089 [Pasteurella multocida subsp. multocida str. HN06]	829.884	4491.33	5.411997339	2.43616113	0.0206	0.597743
PMCN03_0075	hmuT	hemin-binding periplasmic protein HmuT [Pasteurella multocida subsp. multocida str. HN06]	15.1058	76.868	5.088641449	2.34728054	0.04505	0.677116
PMCN03_0669	aroF	phospho-2-dehydro-3-deoxyheptonate aldolase [Pasteurella multocida subsp. multocida str. Pm70]	10.2759	52.0858	5.068733639	2.34162535	0.04415	0.675155
PMCN03_1726	-	hypothetical protein [Pasteurella multocida]	8.71874	42.3246	4.854439976	2.27930487	0.0422	0.667844
PMCN03_0528	alsT	hypothetical protein [Pasteurella multocida]	27.4271	129.448	4.719711526	2.23869868	0.03565	0.639384
PMCN03_2137	exbB	tonB-system energizer ExbB [Pasteurella multocida 36950]	11.4963	53.981	4.69551073	2.23128209	0.03995	0.649737
PMCN03_1033	-	RNA polymerase sigma factor [Pasteurella multocida subsp. multocida str. Pm70]	82.6724	343.937	4.160239693	2.05666665	0.04635	0.681498
PMCN03_1549	-	xylulose kinase [Pasteurella multocida 36950]	80.1412	17.164	0.214171986	-2.2231583	0.03735	0.645668
PMCN03_0994	znuB	hypothetical protein PM0241 [Pasteurella multocida subsp. multocida str. Pm70]	43.8632	9.23052	0.210438819	-2.2485272	0.0311	0.608707
PMCN03_0257	-	CRISPR-associated protein, Csy2 family [Pasteurella multocida]	71.6618	14.9362	0.208426247	-2.2623911	0.04765	0.681498
PMCN03_1763	comF	competence protein F [Pasteurella multocida 36950]	286.919	55.16	0.19224938	-2.3789491	0.0228	0.604352
PMCN03_0583	trpCF	tryptophan biosynthesis protein TrpCF [Pasteurella multocida 36950]	110.122	20.909	0.189871234	-2.3969067	0.02445	0.608707
PMCN03_1279	mogA	molybdenum cofactor biosynthesis protein [Pasteurella multocida 36950]	209.686	38.3915	0.18309043	-2.4493717	0.02625	0.608707
PMCN03_1303	cusC	cation efflux system protein CusC [Pasteurella multocida 36950]	113.961	19.7991	0.173735752	-2.5250334	0.01775	0.597743
PMCN03_1702	-	lipoprotein-2 [Pasteurella multocida 36950]	284.634	47.7505	0.167761055	-2.5755203	0.0159	0.597743
PMCN03_2042	tatD	hypothetical protein PM1675 [Pasteurella multocida subsp. multocida str. Pm70]	122.942	20.2745	0.164911096	-2.6002396	0.0216	0.597743
PMCN03_0173	-	membrane protein [Pasteurella multocida 36950]	239.536	38.5647	0.160997512	-2.6348897	0.0453	0.677116
PMCN03_2034	-	phosphoglycerol transferase [Pasteurella multocida 36950]	293.15	46.8912	0.159956336	-2.64425	0.0472	0.681498
PMCN03_0199	recC	exodeoxyribonuclease V subunit gamma [Pasteurella multocida 36950]	163.289	24.6687	0.151073863	-2.726674	0.0413	0.662132
PMCN03_0955	-	hypothetical protein PM0279 [Pasteurella multocida subsp. multocida str. Pm70]	687.414	102.458	0.149048463	-2.7461466	0.01105	0.597743
PMCN03_1446	-	hypothetical protein PM1826 [Pasteurella multocida subsp. multocida str. Pm70]	1325.21	196.997	0.148653421	-2.7499754	0.03215	0.608707

PMCN03_1466	-	hypothetical protein D11S_0691 [Aggregatibacter actinomycetemcomitans D11S-1]	219.778	32.4795	0.147783218	-2.7584456	0.02725	0.608707
PMCN03_0276	-	hypothetical protein [Pasteurella multocida]	1096.97	161.692	0.147398744	-2.7622039	0.01185	0.597743
PMCN03_1445	-	hypothetical protein PM1827 [Pasteurella multocida subsp. multocida str. Pm70]	2753.19	399.594	0.145138548	-2.7844973	0.0297	0.608707
PMCN03_r17	PMCN03	cell wall-associated hydrolase [Burkholderia multivorans ATCC 17616]	241.991	34.3264	0.141849904	-2.8175629	0.02905	0.608707
PMCN03_0210	-	membrane protein [Pasteurella multocida 36950]	153.446	21.1886	0.138085059	-2.8563709	0.0427	0.667844
PMCN03_1451	ppkA	von Willebrand factor, type A family protein [Pasteurella multocida 36950]	259.401	34.3377	0.132373044	-2.9173187	0.0348	0.639384
PMCN03_0924	miaA	tRNA dimethylallyltransferase [Pasteurella multocida 36950]	46.371	6.13107	0.132217765	-2.9190121	0.0183	0.597743
PMCN03_0956	aceF	dihydrolipoyllysine-residue succinyltransferase [P. multocida 36950]	946.453	123.19	0.130159659	-2.9416457	0.0204	0.597743
PMCN03_0149	wbjC	UDP-2-acetamido-2,6-dideoxy-beta-L-talose 4-dehydrogenase [Pasteurella multocida]	188.233	24.1984	0.128555567	-2.959536	0.03885	0.647332
PMCN03_0874	pgsA	hypothetical protein PM0856 [Pasteurella multocida subsp. multocida str. Pm70]	679.291	85.5169	0.125891407	-2.9897483	0.0348	0.639384
PMCN03_1172	cumB	hypothetical protein [Pasteurella multocida]	998.124	125.516	0.125751911	-2.9913478	0.0394	0.647332
PMCN03_1641	xynC	XynC protein [Pasteurella multocida subsp. multocida str. Pm70]	1462.27	179.787	0.122950618	-3.0238491	0.0176	0.597743
PMCN03_0211	-	arabinose transporter [Pasteurella multocida]	223.238	27.2003	0.121844399	-3.0368882	0.03045	0.608707
PMCN03_0875	uvrC	excinuclease ABC subunit C [Pasteurella multocida]	263.846	31.0385	0.117638698	-3.0875654	0.02835	0.608707
PMCN03_1359	oppB	oligopeptide transporter permease [Pasteurella multocida subsp. multocida str. Pm70]	153.823	18.0498	0.11734136	-3.0912165	0.0062	0.553183
PMCN03_1467	gppA	exopolyphosphatase [Pasteurella multocida]	345.239	40.0377	0.115970965	-3.1081644	0.0272	0.608707
PMCN03_1450	-	ABC transporter-like protein [Pasteurella multocida 36950]	291.322	32.9458	0.113090669	-3.1444482	0.03155	0.608707
PMCN03_0950	galE	UDP-glucose 4-epimerase [Pasteurella multocida]	587.963	60.3426	0.102629927	-3.2844766	0.02015	0.597743
PMCN03_1514	napF	ferredoxin-type NapF family protein [Pasteurella multocida 36950]	151.516	14.958	0.098722247	-3.340481	0.0064	0.553183
PMCN03_0951	ampG	AmpG-like permease [Pasteurella multocida 36950]	221.274	21.2471	0.096021674	-3.3804961	0.02035	0.597743
PMCN03_0046	groS	co-chaperonin GroES [Pasteurella multocida subsp. multocida str. Pm70]	799.421	76.5427	0.095747672	-3.3846188	0.00405	0.53676
PMCN03_1857	dctP	hypothetical protein PM1252 [Pasteurella multocida subsp. multocida str. Pm70]	1066.58	101.102	0.094790827	-3.3991087	0.0102	0.597743
PMCN03_0139	-	putative tyrosine-protein kinase [Pasteurella multocida 36950]	191.066	17.3584	0.090850282	-3.4603652	0.02205	0.597743
PMCN03_1516	dmsC	anaerobic dimethyl sulfoxide reductase chain C [Pasteurella multocida 36950]	438.952	38.2648	0.087173085	-3.5199734	0.0157	0.597743
PMCN03_0586	trpG	anthranilate synthase component TrpG [Pasteurella multocida 36950]	264.453	22.4979	0.08507334	-3.5551491	0.00345	0.53676
PMCN03_0143	arnB	UDP-4-amino-4-deoxy-L-arabinose--oxoglutarate aminotransferase [Pasteurella multocida 36950]	258.299	20.8328	0.080653816	-3.6321134	0.0151	0.597743
PMCN03_0536	torY	cytochrome c-type protein TorY, partial [Pasteurella multocida]	651.28	52.4281	0.080500092	-3.6348658	0.00985	0.597743
PMCN03_1515	-	twin-argninine leader-binding protein DmsD [Pasteurella multocida]	642.509	48.4272	0.075372018	-3.7298272	0.01295	0.597743
PMCN03_0144	speG	GCN5-like N-acetyltransferase (GNAT) domain protein [Pasteurella multocida 36950]	165.243	12.4126	0.075117252	-3.7347119	0.0034	0.53676
PMCN03_0142	rfb	Rfb [Pasteurella multocida]	124.213	9.29307	0.074815599	-3.7405171	0.01365	0.597743
PMCN03_0443	sixA	hypothetical protein PM0441 [Pasteurella multocida subsp. multocida str. Pm70]	878.51	55.1461	0.062772308	-3.9937279	0.0098	0.597743
PMCN03_1517	hybA	anaerobic dimethyl sulfoxide reductase subunit B [Pasteurella multocida 36950]	779.687	48.5998	0.062332449	-4.0038728	0.00965	0.597743
PMCN03_1870	tenA	TENA/THI-4 protein/Coenzyme PQQ biosynthesis protein C family protein [Pasteurella multocida 36950]	627.704	38.6478	0.061570103	-4.0216262	0.0083	0.597743
PMCN03_0719	nrdB	ribonucleotide-diphosphate reductase subunit alpha [Haemophilus somnus 129PT]	2153.38	125.171	0.058127688	-4.1046307	0.0357	0.639384
PMCN03_0133	htpG	chaperone protein HtpG [Pasteurella multocida 36950]	302.13	15.3821	0.05091219	-4.2958451	0.00365	0.53676
PMCN03_1854	-	hypothetical protein PM1249 [Pasteurella multocida subsp. multocida str. Pm70]	1280.1	64.8687	0.050674713	-4.3025902	0.047	0.681498
PMCN03_0824	tnaA	tyrosine phenol-lyase [Pasteurella multocida subsp. multocida str. Pm70]	3311.32	164.493	0.04967596	-4.3313083	0.03045	0.608707
PMCN03_1157	-	ribosome-associated inhibitor A [Pasteurella multocida 36950]	40106.1	1965.66	0.049011497	-4.350736	0.03115	0.608707
PMCN03_0970	pepD	peptidase T [Pasteurella multocida 36950]	619.654	30.3254	0.048939247	-4.3528643	0.0034	0.53676
PMCN03_0222	-	cytochrome c peroxidase [Pasteurella multocida 36950]	1188.8	57.3322	0.048226952	-4.3740166	0.043	0.667844
PMCN03_1866	thiD	hydroxymethylpyrimidine/phosphomethylpyrimidine kinase [Pasteurella multocida 36950]	741.322	32.5244	0.043873512	-4.510506	0.02375	0.608707
PMCN03_0229	-	carboxypeptidase G2 [Pasteurella multocida 36950]	2493.18	96.8521	0.038846814	-4.6860599	0.043	0.667844
PMCN03_0140	amsI	low molecular weight protein-tyrosine-phosphatase AmsI [Pasteurella multocida 36950]	492.913	17.7338	0.035977546	-4.7967594	0.00025	0.497
PMCN03_1865	thiE	thiamine-phosphate pyrophosphorylase [Pasteurella multocida 36950]	1133.78	38.7242	0.034154951	-4.8717615	0.02225	0.597743
PMCN03_0146	rfbP	protein RfbP [Pasteurella multocida subsp. multocida str. Pm70]	192.843	6.47207	0.033561343	-4.8970558	0.0011	0.53676
PMCN03_0535	-	trimethylamine N-oxide reductase I catalytic subunit [Pasteurella multocida]	1018.02	31.9831	0.031416966	-4.9923123	0.01985	0.597743
PMCN03_0141	-	polysaccharide export protein Wza [Pasteurella multocida 36950]	390.809	12.0333	0.030790744	-5.0213594	0.00215	0.53676
PMCN03_1518	bisC	dimethyl sulfoxide reductase subunit A [Haemophilus influenzae]	644.269	16.4685	0.025561528	-5.2898821	0.02145	0.597743
PMCN03_1634	glpQ	glycerophosphoryl diester phosphodiesterase [Pasteurella multocida 36950]	1426.76	33.5434	0.023510191	-5.4105699	0.02135	0.597743
PMCN03_1869	thiY	thiamine biosynthesis protein [Pasteurella multocida 36950]	2749.69	41.3504	0.015038204	-6.0552239	0.0086	0.597743
PMCN03_2089	pdxT	glutamine amidotransferase subunit PdxT [Pasteurella multocida 36950]	46599.4	114.003	0.002446448	-8.6750958	0.01835	0.597743

Table S4. Giant checkerboard assay of florfenicol (FFC) and thiamphenicol (TAP) against *P. multocida*, *S. suis,* and *S. hyicus*.

Strain	Case NO.	Antibiotic MIC alone (mg/L)		Antibiotic MIC in combination (mg/L)	FIC index	Synergy	
		FFC	TAP	FFC+TAP		BSAC	Time-kill curve
S. hyicus	CM144-2B	64	1024	4+384	0.438	Yes	Yes
S. suis	BL25-2	2	4	0.25+1.5	0.500	Yes	Yes
S. suis	CY13005-1	2	8	0.25+3	0.500	Yes	Yes
P. multocida	0425	1	1	0.125+0.375	0.500	Yes	Yes
P. multocida	0965	32	512	4+256	0.625	No	Yes
P. multocida	101-035	16	512	0.5+192	0.406	Yes	Yes
P. multocida	101-086	16	1024	1+384	0.438	Yes	Yes
P. multocida	102-033	16	1024	1+512	0.563	No	Yes
P. multocida	D7	0.5	1	0.016+0.375	0.406	Yes	Yes
P. multocida	D13	0.5	1	0.031+0.375	0.438	Yes	Yes
P. multocida	A8	0.5	1	0.063+0.375	0.500	Yes	Yes
P. multocida	D3	0.5	1	0.063+0.5	0.625	No	Yes
P. multocida	D4	0.5	1	0.063+0.375	0.500	Yes	Yes

BSAC, British Society for Antimicrobial Chemotherapy recommendation;
FICI, fractional inhibitory concentration index
